# Effect of Zn and Ca Addition on Microstructure and Strength at Room Temperature of As-Cast and As-Extruded Mg-Sn Alloys

**DOI:** 10.3390/ma11091490

**Published:** 2018-08-21

**Authors:** Yang Zhang, Leipeng Song, Xiaoyang Chen, Yalin Lu, Xiaoping Li

**Affiliations:** Key lab of advanced material design and additive manufacturing of Jiangsu Province, Jiangsu University of Technology, Changzhou 213001, China; zhangyang@jsut.edu.cn (Y.Z.); songleipeng0130@163.com (L.S.); luyalin@163.com (Y.L.); lxp118@jsut.edu.cn (X.L.)

**Keywords:** Mg-Sn alloy, as-cast, hot extrusion, microstructure, strength at room temperature

## Abstract

In this study, the effect of Zn and Ca addition on microstructure and strength at room temperature of Mg-Sn alloys was investigated by comparison of Mg-6Sn, Mg-6Sn-2Zn, and Mg-6Sn-2Zn-1Ca alloys in as-cast and as-extruded states. In the as-cast samples, α-Mg and Mg_2_Sn phases were the main phases of Mg-6Sn and Mg-6Sn-2Zn alloys, while the CaMgSn phase was formed in Mg-6Sn-2Zn-1Ca alloy due to the addition of the Ca element. Mg_2_Sn phase dissolved into the matrix during homogenization while CaMgSn phase remained. Incomplete dynamic recrystallization (DRX) took place in these alloys during hot extrusion. Fine Mg_2_Sn precipitates were observed in α-Mg matrix of as-extruded samples. Zn showed little influence on microstructure, whereas Ca reduced the volume fraction of un-DRXed grains and increased the size of DRXed grains. As-extruded Mg-Sn alloys exhibited typical fiber texture. The strength at room temperature of Mg-Sn alloys improved significantly after hot extrusion. The addition of Zn element was beneficial to the strength at room temperature of the Mg-6Sn alloy, while the further addition of Ca element was harmful to the strength. Among these alloys, the Mg-6Sn-2Zn alloy exhibited the best strength at room temperature in both as-cast and as-extruded states.

## 1. Introduction

Mg alloys have drawn attention in the past decades due to its advantage of having high strength–weight ratio, which helps its application in the transportation industry with improved fuel efficiency and reduced pollutant emission [1,2]. Mg-Al alloys, such as the AZ and AM series, have been widely used due to their good corrosion resistance and qualified strength at room temperature. However, the melting point of the eutectic phase, Mg_17_Al_12_ phase, is only 437 °C, which provides Mg-Al alloys a relatively narrow application for a temperature range [3,4]. Over the past few years, Mg-rare earth (RE) alloys have shown good heat resistance and have been utilized successfully in the aerospace field. RE elements are efficient in improving the heat resistance of Mg alloys [5,6]. But the price of RE elements is so high that it restricts further application of Mg-RE alloys. Recently, Mg-Sn alloys have received increasing investigation and interest as a novel heat-resistant Mg alloy, whose eutectic phase, Mg_2_Sn phase, has a high melting point at 770 °C [7,8,9]. The microstructure and strength at room temperature of novel Mg-Sn alloys is an emergent task to study.

Single or multiple elemental alloying has been used to modify the strength at room temperature of Mg-Sn alloys. For example, Tang et al. [10] found the addition of Zn caused the formation of Mg_2_Sn and MgZn precipitates in indirectly extruded samples and led to better strength at room temperature and lower yield asymmetry. Khalilpour et al. [11] found the addition of Ca brought about the formation of uniformly distributed CaMgSn and Mg_2_Ca phases in as-cast samples and generated good creep resistance. Pan et al. [12] compared the effect of Ca and Ca-Zn alloying in Mg-Sn alloys. In both situations, high strength and ductility were obtained. Though the multiple alloying of Ca and Zn was beneficial to elongation, the strength was reduced. However, Chang et al. [13] found the addition of Ca into Mg-Sn-Zn alloy improved the strength and ductility simultaneously. Therefore, the effect of Ca and Zn elements on strength at room temperature of Mg-Sn alloys is not clear and needs to be further studied.

In this study, to clarify the effect of Zn and Ca addition to Mg-Sn, Mg-6Sn, Mg-6Sn-2Zn, and Mg-6Sn-2Zn-1Ca (wt %) alloys are designed for comparison. The alloys were prepared by permanent mold casting and then hot extruded. The microstructure and strength at room temperature of as-cast and as-extruded Mg-6Sn, Mg-6Sn-2Zn, and Mg-6Sn-2Zn-1Ca alloys were investigated systematically. The microstructure evolution and strengthening mechanism are discussed.

## 2. Materials and Methods

The alloy ingots with nominal compositions of Mg-6Sn, Mg-6Sn-2Zn, and Mg-6Sn-2Zn-1Ca (wt %) were prepared by melting of commercial pure Mg, Sn, Zn (99.9 wt %) and Mg-30Ca (wt %) master alloy. The chemical composition of the alloys was determined by an inductively coupled plasma analyzer (ICP, Optima 8000DV, PerkinElmer, Waltham, MA, USA) and the exact chemical composition was Mg-5.87Sn, Mg-5.91Sn-1.84Zn, and Mg-5.75Sn-2.05Zn-0.83Ca (wt %), respectively. The melt was poured into a permanent mold preheated to 200 °C. Mixed atmosphere of SF_6_ (1 vol %) and CO_2_ (99 vol %) was used to protect the melting process. The cast ingots were homogenized at 480 °C for 12 h and then quenched into cold water. The hot extrusion was carried out at 300 °C with an extrusion ratio of 9:1.

**Figure 1:**
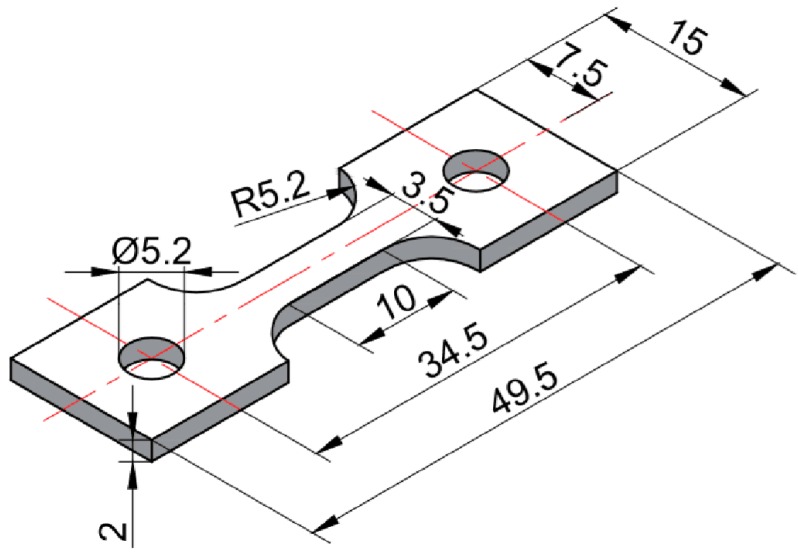
The dimensions of tensile specimens used in this study (unit: mm).

X-ray diffraction (XRD, Xpert Powder, Mavern Panalytical, Almelo, The Netherlands) was used to characterize the phase composition of as-cast Mg-Sn alloys. Optical microscope (OM, Zeiss Primotech, Berlin, Germany), scanning electron microscope (SEM, Simga 500 VP, Zeiss, Oberkochen, Germany) equipped with energy dispersive spectrometer (EDS, Inca, Oxford Instruments, Oxford, UK) and a transmission electron microscope (TEM, Talos, Thermo Fisher Scientific, Hillsboro, OR, USA) were used for microstructure characterization. Electron backscattered diffraction (EBSD, Inca, Oxford Instruments, Oxford, UK) was used for texture characterization. The grain size of as-extruded samples was determined with the data obtained by EBSD. The grain size of as-cast and as-homogenized samples were determined by the linear intercept method. Tensile specimens were cut from as-cast ingots and the extruded bars parallel to the extrusion direction. The dimensions of tensile specimens can be seen in Figure 1, with gage length of 10 mm, width of 3.5 mm, and thickness of 2.0 mm. A universal testing machine (CMT-5205, Wance, Shenzhen, China) was used for tensile test with a strain rate of 1.67 × 10^−3^ s^−1^ at ambient temperature according to ISO6892-1:2009 [14]. The strain measurement was done with an extensometer. For each sample, at least three specimens were tested and the average was taken. 

## 3. Results

**Figure 2:**
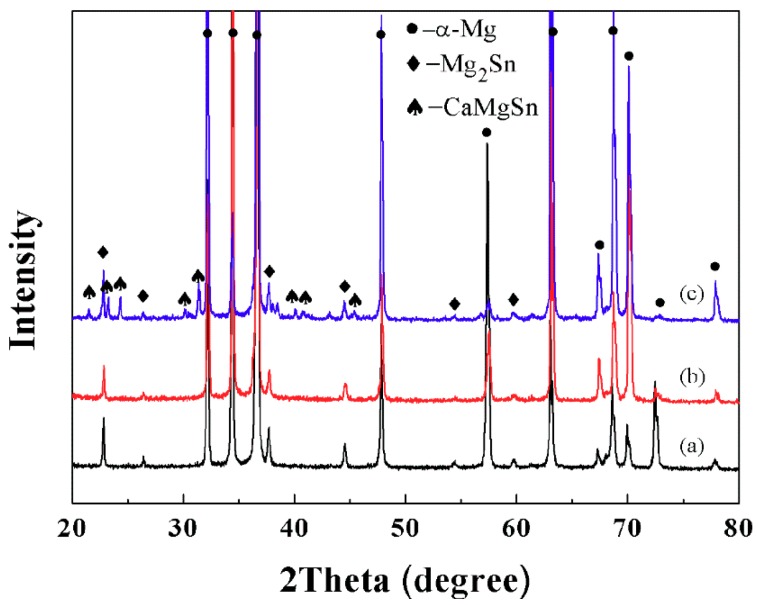
XRD patterns and analysis results of as-cast Mg-Sn alloys: (**a**) Mg-6Sn; (**b**) Mg-6Sn-2Zn; and (**c**) Mg-6Sn-2Zn-1Ca.

Figure 2 shows XRD patterns and analysis results of as-cast Mg-6Sn, Mg-6Sn-2Zn, and Mg-6Sn-2Zn-1Ca alloys. As seen in Figure 2, XRD patterns of both Mg-6Sn and Mg-6Sn-2Zn alloys are almost the same. The main phases are α-Mg phase and Mg_2_Sn phase, confirming the addition of Zn element (2 wt %) had little influence on phase composition of the Mg-6Sn alloy. In the pattern of the Mg-6Sn-2Zn-1Ca alloy, several peaks corresponding to the CaMgSn phase exist. It indicates that the addition of Ca element (1 wt %) can influence the phase composition of Mg-6Sn-2Zn alloy.

**Figure 3:**
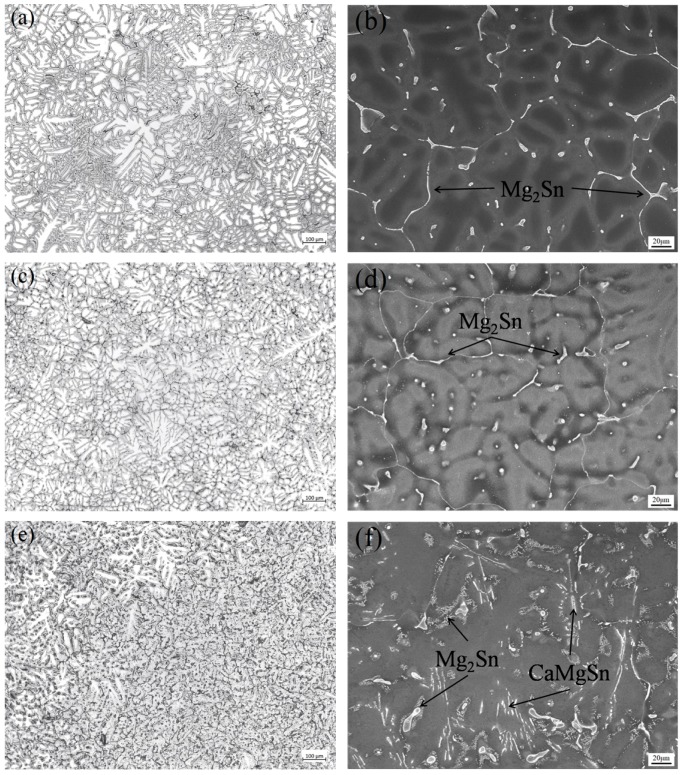
Optical microstructure and SEM images of as-cast samples: (**a**,**b**) Mg-6Sn; (**c**,**d**) Mg-6Sn-2Zn; and (**e**,**f**) Mg-6Sn-2Zn-1Ca.

Figure 3 shows the optical microstructure and SEM images of as-cast Mg-6Sn, Mg-6Sn-2Zn, and Mg-6Sn-2Zn-1Ca alloys. Figure 3a,c,e are the optical images of the cast samples. It is shown that the matrix of as-cast samples all consist of dendritic α-Mg phase. With the addition of the Zn element and further addition of the Ca element, α-Mg dendrites were refined gradually. The average grain size of as-cast Mg-6Sn, Mg-6Sn-2Zn, and Mg-6Sn-2Zn-1Ca alloys was 175 ± 33 μm, 106 ± 21 μm, and 82 ± 9 μm, respectively. As for the eutectic Mg_2_Sn phase, it exhibited two different morphologies, one was isolated-particle-like and the other was connected-network-like, as shown in Figure 3b,d. There was no fundamental difference in the microstructure between as-cast Mg-6Sn and Mg-6Sn-2Zn alloys. As seen in Figure 3f, with the addition of Ca element, the morphologies of second phases in the as-cast Mg-6Sn-2Zn-1Ca alloy were different from those in the Mg-6Sn and Mg-6Sn-2Zn alloys. The volume fraction of eutectic Mg_2_Sn phase decreased obviously. Numerous fine Mg_2_Sn phases which were supposed to be precipitated after solidification were found near the grain boundaries. Also, a large number of lamellar phases were observed. According to EDS results, the lamellar phase as determined to be CaMgSn phase.

**Figure 4:**
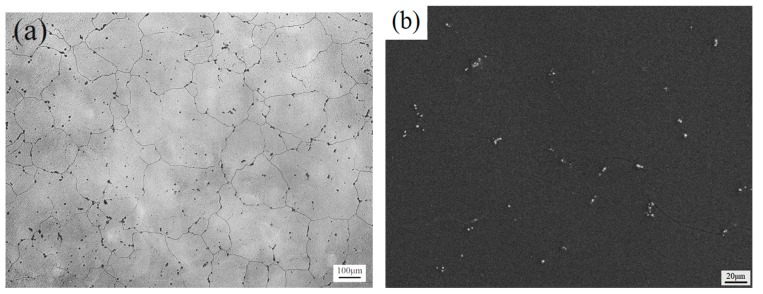

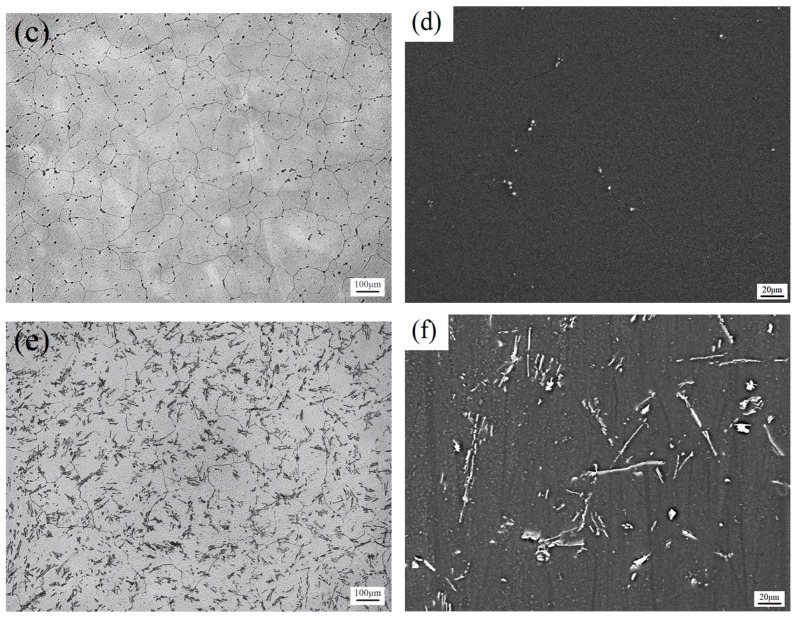
Optical microstructure and SEM images of as-homogenized samples: (**a**,**b**) Mg-6Sn; (**c**,**d**) Mg-6Sn-2Zn; and (**e**,**f**) Mg-6Sn-2Zn-1Ca.

Figure 4 shows the optical microstructure and SEM images of as-homogenized Mg-6Sn, Mg-6Sn-2Zn, and Mg-6Sn-2Zn-1Ca alloys. After being homogenized at 480 °C for 12 h, α-Mg dendrites turned to be nearly equiaxed. The coarsening of α-Mg grains was also observed. The average grain size of as-cast Mg-6Sn, Mg-6Sn-2Zn, and Mg-6Sn-2Zn-1Ca alloys was increased to 203 ± 19 μm, 144 ± 23 μm, and 138 ± 26 μm, respectively. Most of Mg_2_Sn phase in Mg-6Sn and Mg-6Sn-2Zn alloys dissolved into α-Mg matrix, and only several small Mg_2_Sn particles were observed at grain boundaries (seen from Figure 4a–d). However, in the as-homogenized Mg-6Sn-2Zn-1Ca alloy, although eutectic and precipitated Mg_2_Sn phase almost dissolved into α-Mg matrix, CaMgSn phase remained, as seen in Figure 4e,f. The shape of the CaMgSn phase was almost the same as that in the as-cast state, which indicates that the thermal stability of the CaMgSn phase was better than the Mg_2_Sn phase.

**Figure 5:**
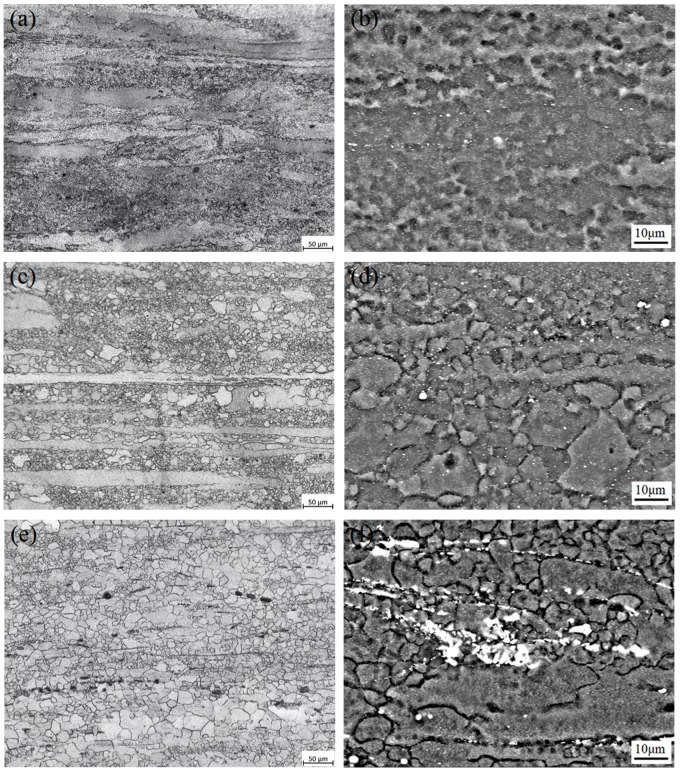
Optical microstructure and SEM images of as-extruded samples: (**a**,**b**) Mg-6Sn; (**c**,**d**) Mg-6Sn-2Zn; and (**e**,**f**) Mg-6Sn-2Zn-1Ca.

Figure 5 displays the optical microstructure and SEM images of as-extruded Mg-6Sn, Mg-6Sn-2Zn, and Mg-6Sn-2Zn-1Ca alloys (along the extrusion direction). As seen in Figure 5a,c,e, incomplete dynamic recrystallization (DRX) takes place in the as-extruded Mg-Sn alloys and the microstructure was obviously refined. The microstructure of the as-extruded Mg-Sn alloys mainly consisted of refined DRX grains and coarse elongated grains. As shown in Figure 5b,d,f, dense precipitates were observed in the α-Mg matrix in the as-extruded Mg-Sn alloys. According to Chang et al. [13], these fine precipitates were probably of the Mg_2_Sn phase, which form dynamically during hot extrusion. Furthermore, as seen in Figure 5f, the needle-like CaMgSn phase in the as-homogenized Mg-6Sn-2Zn-1Ca alloy was broken into particles and distributed along the extrusion direction. 

**Figure 6:**
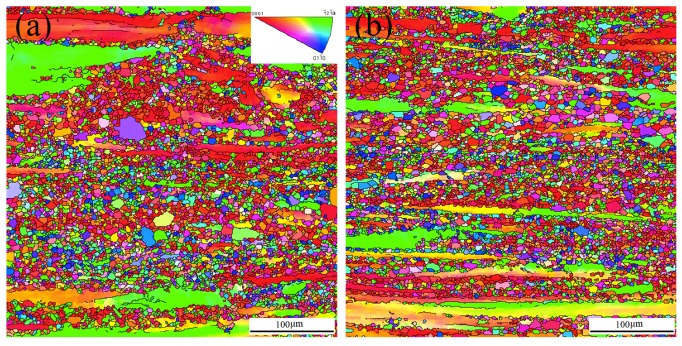

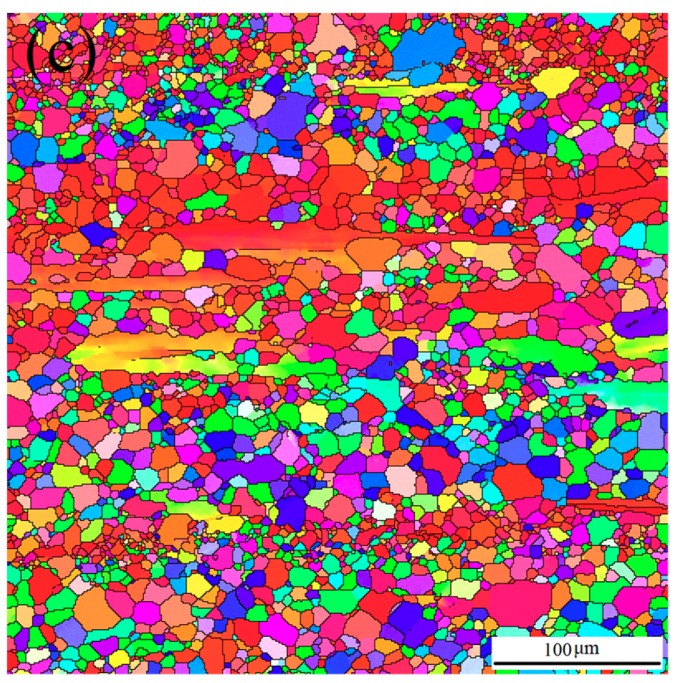
Inverse pole figure maps of as-extruded Mg-Sn alloys obtained by electron backscattered diffraction (EBSD, along the extrusion direction): (**a**) Mg-6Sn; (**b**) Mg-6Sn-2Zn; and (**c**) Mg-6Sn-2Zn-1Ca.

Figure 6 shows the inverse pole figure maps of the as-extruded Mg-6Sn, Mg-6Sn-2Zn, and Mg-6Sn-2Zn-1Ca alloys obtained by EBSD (along the extrusion direction). The EBSD maps show that DRX takes place in the Mg-Sn alloys during hot extrusion, but incompletely. The fine equiaxed grains are DRX grains, and the coarse un-DRX grains were elongated along the extrusion direction. The addition of the Zn element showed little influence on the microstructure of the as-extruded sample and the size and volume fraction of DRX grains were almost the same in the as-extruded Mg-6Sn and Mg-6Sn-2Zn alloys. However, the addition of Ca element remarkably reduced the volume fraction of un-DRX grains in the as-extruded Mg-6Sn-2Zn-1Ca alloy. On the other hand, DRX grains in the as-extruded Mg-6Sn-2Zn-1Ca alloy were coarser than those in the Mg-6Sn and Mg-6Sn-2Zn alloys. The differences in grain size were confirmed by quantitative analysis. The average grain size of the as-extruded Mg-6Sn, Mg-6Sn-2Zn, and Mg-6Sn-2Zn-1Ca alloys was about 4.6 ± 2.9 μm, 4.6 ± 2.8 μm, and 6.4 ± 3.5 μm, respectively. Compared to the as-extruded Mg-6Sn and Mg-6Sn-2Zn samples, the average grain size in the Mg-6Sn-2Zn-1Ca sample increased by about 39 %.

**Figure 7:**
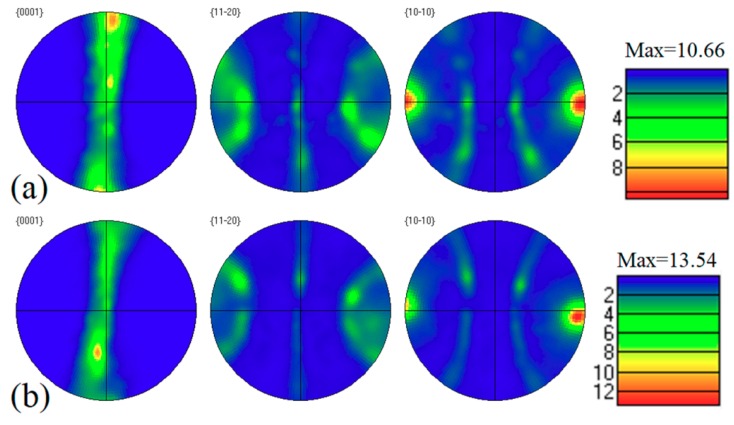

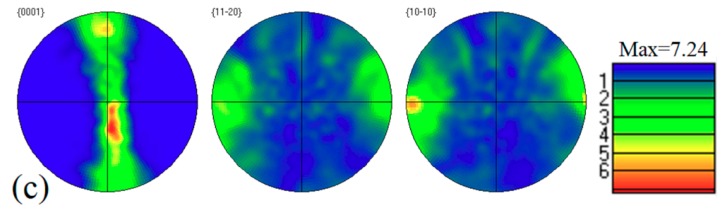
Pole figures of as-extruded Mg-Sn alloys obtained by EBSD (along the extrusion direction): (**a**) Mg-6Sn; (**b**) Mg-6Sn-2Zn; and (**c**) Mg-6Sn-2Zn-1Ca.

Figure 7 shows the corresponding pole figures of the as-extruded Mg-6Sn, Mg-6Sn-2Zn, and Mg-6Sn-2Zn-1Ca alloys obtained by EBSD (along the extrusion direction). As shown in Figure 7, the as-extruded Mg-Sn alloys exhibited typical fiber texture. The {0001} basal plane of Mg was aligned along the extrusion direction with its <10–10> direction parallel to the extrusion direction. This kind of texture is in accordance with the usual extruded structure in Mg alloys. As for the texture intensity, the addition of Zn element led to an increase from 10.66 of the as-extruded Mg-6Sn alloy to 13.54 of the as-extruded Mg-6Sn-2Zn alloy. However, the further addition of Ca element led to a significant decrease from 13.54 of the as-extruded Mg-6Sn-2Zn alloy to 7.24 of the as-extruded Mg-6Sn-2Zn-1Ca alloy.

**Figure 8:**
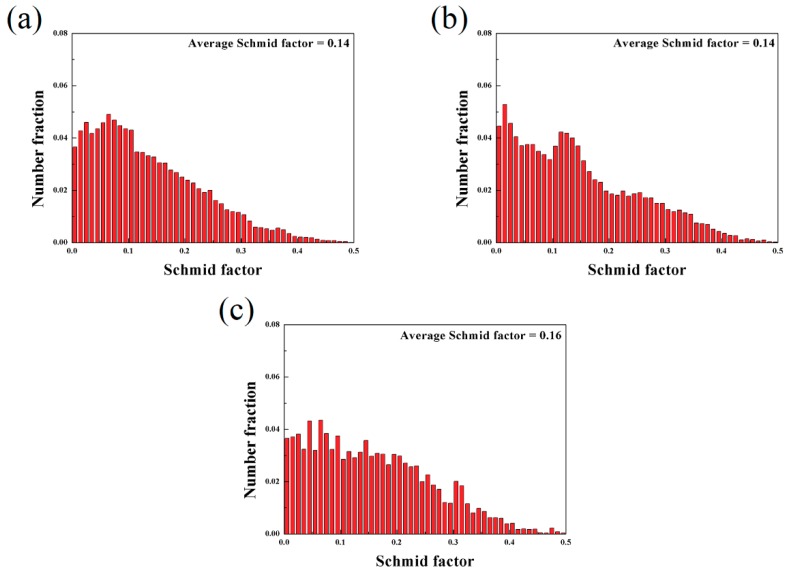
The distribution of Schmid factor of the as-extruded Mg-Sn alloys: (**a**) Mg-6Sn; (**b**) Mg-6Sn-2Zn; and (**c**) Mg-6Sn-2Zn-1Ca.

Figure 8 shows the distribution of Schmid factor of the as-extruded Mg-Sn alloys obtained by EBSD. It is found in Figure 8 that the distribution of Schmid factor of the as-extruded Mg-6Sn, Mg-6Sn-2Zn, and Mg-6Sn-2Zn-1Ca alloys showed little difference. The average Schmid factor of the as-extruded Mg-Sn alloys increased slightly, from 0.14 in the Mg-6Sn alloy to 0.16 in the Mg-6Sn-2Zn-1Ca alloy.

**Figure 9:**
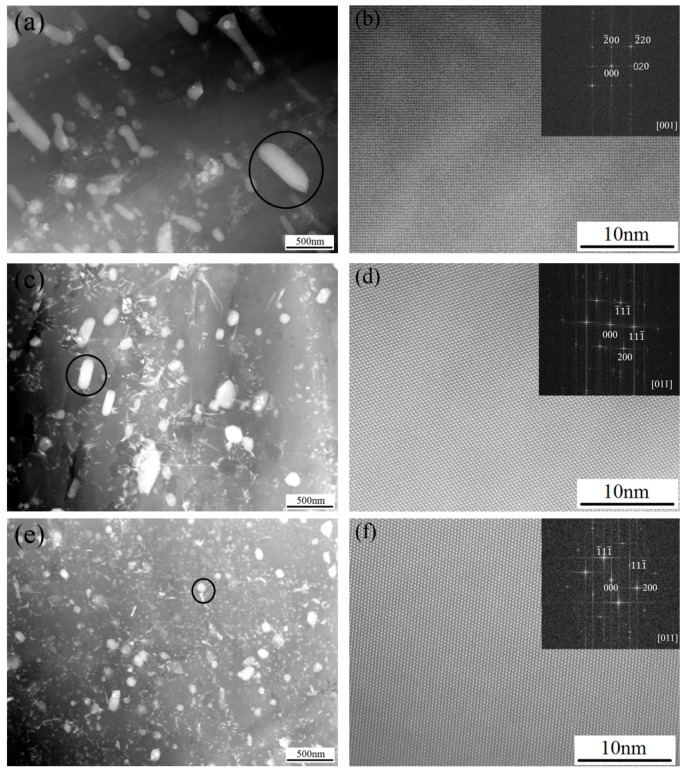
TEM results of as-extruded Mg-Sn alloys: (**a**,**b**) Mg-6Sn; (**c**,**d**) Mg-6Sn-2Zn; and (**e**,**f**) Mg-6Sn-2Zn-1Ca.

To identify the fine precipitates in the as-extruded Mg-6Sn, Mg-6Sn-2Zn, and Mg-6Sn-2Zn-1Ca alloys, TEM observation was conducted. As shown in Figure 9a, there were many rod-like precipitates in the as-extruded Mg-6Sn alloy. As shown in Figure 9c, the precipitates in the as-extruded Mg-6Sn-2Zn alloy were refined and the length–width ratio was also reduced. As shown in Figure 9e, the precipitates in the as-extruded Mg-6Sn-2Zn-1Ca alloy was further refined and the rod-like shape turned to be block-like or spherical shaped. The high resolution TEM images in Figure 9b,d,f was taken from the circled area in Figure 9a,c,e, respectively, and corresponding Fourier transformed patterns were inserted. It was confirmed that the precipitates in the as-extruded alloys were Mg_2_Sn phases.

**Figure 10:**
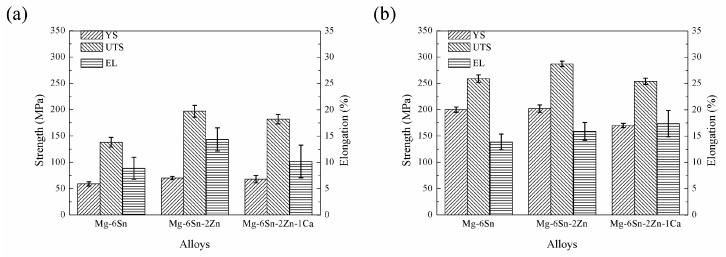
Strength and elongation at room temperature of as-cast and as-extruded Mg-Sn alloys: (**a**) as-cast and (**b**) as-extruded.

Figure 10 shows the influence of Zn and Ca addition on strength at room temperature of as-cast and as-extruded Mg-Sn alloys. For as-cast samples, yield strength (YS), ultimate tensile strength (UTS), and elongation (EL) of the Mg-6Sn alloy were 59 ± 4 MPa, 138 ± 9 MPa, and 8.9 ± 2.1%, respectively. With the addition of Zn element, YS, UTS, and EL of Mg-6Sn-2Zn alloy were improved to 70 ± 3 MPa, 197 ± 11 MPa, and 14.4 ± 1.7%, respectively. However, further addition of Ca element was harmful to strength at room temperature, and YS, UTS, and EL of Mg-6Sn-2Zn-1Ca alloy were reduced to 68 ± 7 MPa, 182 ± 9 MPa, and 10.2 ± 3.1%, respectively. For the as-extruded samples, strength at room temperature was improved significantly when compared to the as-cast samples. The YS, UTS, and EL of the Mg-Sn alloys were improved by 150.2–239.8%, 39.7–87.6%, and 10.4–70.6%. Similar to the as-cast state, the as-extruded Mg-6Sn-2Zn alloy exhibited the best strength at room temperature with a YS of 202 ± 7 MPa, UTS of 287 ± 5 MPa, and EL of 15.9 ± 1.7%. Though the Mg-6Sn-2Zn-1Ca alloy presented the highest EL of 17.4 ± 2.5%, its YS and UTS were lower than those of the as-extruded Mg-6Sn and Mg-6Sn-2Zn alloys.

## 4. Discussion

As shown in Figure 2 and Figure 3, the addition of Zn and Ca elements significantly influenced the as-cast microstructure of Mg-Sn alloys, including α-Mg grain size and phase composition. Firstly, it is apparent that the grains of α-Mg were refined with the addition of Zn element and further addition of Ca element gradually. Usually, the grain refinement of the as-cast Mg alloys could be caused by promoting nucleation and/or restricting growth of α-Mg grains [15,16]. In this study, the alloy element, neither Zn nor Ca, could act as nuclei in Mg melt. Therefore, the grain refinement in as-cast Mg-Sn alloys was merely attributed to the growth restriction effect of Zn and Ca elements. Zn and Ca elements, as the solute elements in Mg melt, could control the growth of nucleated grains via generating constitutional undercooling and inhibiting the diffusion of solute at the solid/liquid interface. According to the calculation [17], both Zn and Ca elements had a relative high growth restriction factor (GRF) in Mg alloys, which indicated that Zn and Ca elements had strong growth restriction ability. Secondly, the addition of Zn and Ca elements also influenced the form and relative content of secondary phases in the as-cast Mg-Sn alloys. According to the Mg-Sn binary equilibrium phase diagram, the eutectic reaction occurred at 561.2 °C and Mg_2_Sn phase forms. The addition of the Zn element did not lead to the formation of any new phases, which is in accordance with the previous report by Mahallawya et al. [18]. While Zn addition increases, the secondary phase composition becomes sensitive to the ratio of Zn/Sn. With a Zn/Sn ratio of 0.6, (α-Mg + Mg_4_Zn_7_) eutectic phase was observed in as-cast Mg-5Sn-3Zn (wt %) alloy [13]. However, in this study, the Zn/Sn ratio is too low to form Mg_4_Zn_7_ and no (α-Mg + Mg_4_Zn_7_) eutectic phase as found. CaMgSn phase was another secondary phase, which as frequently observed in the as-cast Mg-Sn-Ca-(Zn) alloys, as well as in the Mg-6Sn-2Zn-1Ca alloy in this study. Due to the low relative atomic mass of Ca element (40.0) and the high relative atomic mass of Sn element (118.7), the formation of CaMgSn phase consumed a large amount of the Sn element. Therefore, the relative contents of Mg_2_Sn phase in the as-cast Mg-6Sn and Mg-6Sn-2Zn alloys were higher than that in the Mg-6Sn-2Zn-1Ca alloy. Thirdly, additional Ca influenced the solution ability of Sn in α-Mg dendrite. Fine Mg_2_Sn precipitates were also observed around grain boundaries in the as-cast Mg-6Sn-2Zn-1Ca alloy. The precipitation of Mg_2_Sn phase indicates that the solid solubility of the Sn element in Mg-Sn alloy was reduced with the addition of Ca element.

As shown in Figure 10a, the microstructure evolution of as-cast Mg-Sn alloys induced by the addition of Zn and Ca elements results in the variation in strength at room temperature. All as-cast Mg-Sn alloys in this study exhibited relatively low strength. Compared to the Mg-6Sn alloy, the Mg-6Sn-2Zn alloy exhibited higher YS and UTS, which were improved by 19.7% and 43.0%, respectively. The improvement in strength is mainly attributed to not only grain refinement but also solid solution strengthening. The secondary phases do not seem to provide a positive influence on YS and UTS. The strengthening effect of the coarse Mg_2_Sn phase which locates at grain boundaries was limited. Further, addition of Ca element led to a slight decrease in strength of the as-cast Mg-6Sn-2Zn-1Ca alloy due to the formation of CaMgSn phase. The formation of CaMgSn phase consumes a large amount of Sn element, which weakens solid solution strengthening of Sn in th ematrix phase. As for ductility of the as-cast Mg-Sn alloys, due to the grain refinement by addition of the Zn element, EL of Mg-6Sn-2Zn was significantly improved by 61.8%. Although the addition of the Ca element led to the further grain refinement in the as-cast Mg-6Sn-2Zn-1Ca alloy, and its EL deteriorated compared to the Mg-6Sn-2Zn alloy. The brittle needle-like CaMgSn phase may be responsible for the reduction in ductility of the as-cast Mg-6Sn-2Zn-1Ca alloy. It is easy to induce stress concentration around the speculate-shaped phase and form microcracks near the CaMgSn phase during deformation.

The as-cast microstructure of Mg-Sn alloys is refined obviously during hot extrusion, proved in Figure 5 and Figure 6. A large volume of DRX grains can be seen in all three samples. The values of average grain size of DRX grains in Mg-6Sn and Mg-6Sn-2Zn alloys were close to each other, so were the volume fractions of un-DRX grains. This phenomenon confirms that the addition of the Zn element into Mg-6Sn alloy shows little influence on the as-extruded microstructure. However, the further addition of Ca element into Mg-6Sn-2Zn alloy leads to more and larger DRX grains. The increase in volume fraction and average grain size of DRX grains are attributed to the formation of the CaMgSn phase and the reduction of Mg_2_Sn precipitates. The CaMgSn phase broke into particles during hot extrusion. The presence of the broken CaMgSn phase can promote DRX via a particle-stimulated nucleation (PSN) mechanism [19,20]. Therefore, DRX is enhanced in the as-extruded Mg-6Sn-2Zn-1Ca alloy. Since a large amount of theSn element is consumed in the CaMgSn phase, the formation of Mg_2_Sn precipitates was restricted during extrusion.

The dynamic Mg_2_Sn precipitates play a comprehensive role in DRX of Mg alloys. First, the precipitates can suppress DRX within the original grains through inhibiting the dislocation motion and keepin coarse grains un-DRXed [21]. Thus, the un-DRXed grains were mainly observed in as-extruded Mg-6Sn and Mg-6Sn-2Zn alloys with more Mg_2_Sn precipitates. Second, the precipitates can suppress the growth of DRXed grains, since the plastic deformation introduces a mass of dislocations, where the precipitates tend to nucleate at [22,23]. Considering the two aspects of the Mg_2_Sn phase, the less un-DRX grains and larger DRX grains in the as-extruded Mg-6Sn-2Zn-1Ca alloy can be explained.

Although the addition of Ca promotes DRX of as-extruded Mg-6Sn-2Zn-1Ca alloy in this study, the pole figures in Figure 7 indicates that the texture was little influenced except for the intensity. The texture intensity decreased significantly with the addition of Ca element. The Ca element had a weakening effect on the texture of the extruded sheets, as pointed out by Ding et al. [24], because of the reduction of the c/a ratio and the decrease of stacking fault energy (SFE). In this study, the reduction of un-DRXed grains was also one of the reasons for texture weakening. After extrusion, DRXed grains exhibited relatively random texture and un-DRXed grains exhibited strong basal texture [25]. The lower the volume fraction of un-DRXed grains, the weaker the texture intensity would be.

As shown in Figure 10, the comparison between as-cast and as-extruded Mg-Sn alloys confirms that the strength at room temperature of Mg-Sn alloys is improved by hot extrusion significantly. The strengthening effect includes the grain refinement through DRX, the precipitation of fine Mg_2_Sn phase, and the formation of texture. The classic Hall–Petch relation points that YS is proportional to *d*^−1/2^, where *d* is the average grain size. According to this relationship, the average size of DRXed grains of as-extruded Mg-6Sn and Mg-6Sn-2Zn alloys is almost the same and should provide similar YS. The average size of DRXed grains of as-extruded Mg-6Sn-2Zn-1Ca alloy was larger and would provide a relatively lower YS. The conductions are in coincidence with the results in Figure 9, in which as-extruded Mg-6Sn and Mg-6Sn-2Zn alloys exhibited close YS, and the YS of as-extruded Mg-6Sn-2Zn-1Ca alloy was reduced. The precipitation strengthening from Mg_2_Sn precipitates are also important. The dispersion of Mg_2_Sn precipitates in as-extruded samples can enhance YS substantially. Compared to as-extruded Mg-6Sn-2Zn-1Ca alloy, as-extruded Mg-6Sn and Mg-6Sn-2Zn alloys have more Mg_2_Sn precipitates and higher YS. The effect of un-DRXed grains should not be neglected. The coarse un-DRXed grains with strong basal texture contribute to the high YS while the fine DRXed grains mainly contribute to the ductility [26,27]. As shown in Figure 6a,b, as-extruded Mg-6Sn and Mg-6Sn-2Zn alloys exhibit typical bimodal grain structure which consists of coarse un-DRXed grains and fine DRXed grains while as-extruded Mg-6Sn-2Zn-1Ca alloy almost consists of completely of DRXed grains. Therefore, as-extruded Mg-6Sn-2Zn-1Ca alloy exhibits higher EL than as-extruded Mg-6Sn and Mg-6Sn-2Zn alloys due to the enhanced DRX and reduced texture intensity.

## 5. Conclusions

In this study, the effect of Zn and Ca addition on microstructure and strength at room temperature of Mg-Sn series alloy was investigated by comparison of as-cast and as-extruded Mg-6Sn, Mg-6Sn-2Zn, and Mg-6Sn-2Zn-1Ca alloys and the main conclusions are listed as follows.

(1) Microstructure of as-cast Mg-6Sn alloys was refined with the addition of Zn and Ca elements. Both Mg-6Sn and Mg-6Sn-2Zn alloys consisted of α-Mg and Mg_2_Sn phases. The addition of the Ca element led to the formation of CaMgSn phase in Mg-6Sn-2Zn-1Ca alloy. After homogenization at 480 °C for 12 h, the Mg_2_Sn phase dissolved into the matrix while the CaMgSn phase remained.
(2) Fine Mg_2_Sn precipitates were observed in the α-Mg matrix of the as-extruded Mg-Sn alloys. The formation of the CaMgSn phase consumed a large amount of the Sn element, and Mg_2_Sn precipitates in the as-extruded Mg-6Sn-2Zn-1Ca alloy were reduced compared to the as-extruded Mg-6Sn and Mg-6Sn-2Zn alloys in consequence.
(3) Incomplete DRX takes place in Mg-Sn alloys during hot extrusion. The addition of the Zn element showed little influence on microstructure of the as-extruded Mg-6Sn alloy. The addition of the Ca element reduced the volume fraction of un-DRXed grains, while the DRXed grains were coarsened. As-extruded Mg-Sn alloys exhibited typical fiber texture. The addition of Zn and Ca elements had little influence on the texture of as-extruded Mg-Sn alloys except for the texture intensity.
(4) The strength at room temperature of Mg-Sn alloys was improved significantly after hot extrusion. The strengthening effect included the grain refinement through DRX, the precipitation of fine Mg_2_Sn phase during hot extrusion, as well as the formation of texture. The addition of the Zn element was beneficial to the strength at room temperature of Mg-6Sn alloy while the further addition of Ca element was harmful to the strength. Mg-6Sn-2Zn alloy exhibited the best strength at room temperature at both as-cast and as-extruded states.
